# A case of SLE with COVID-19 and multiple infections

**DOI:** 10.1515/med-2020-0238

**Published:** 2020-10-14

**Authors:** Ruoqi Ning, Silu Meng, Fangxu Tang, Chong Yu, Dong Xu, Xiaofang Luo, Haiying Sun

**Affiliations:** Department of Obstetrics and Gynecology, Tongji Hospital, Tongji Medical College, Huazhong University of Science and Technology, Wuhan, 430030, Hubei, China; Department of Nephrology, Tongji Hospital, Tongji Medical College, Huazhong University of Science and Technology, Wuhan, 430030, Hubei, China; Department of Infectious Diseases, Tongji Hospital, Tongji Medical College, Huazhong University of Science and Technology, Wuhan, 430030, Hubei, China; Department of Obstetrics and Gynecology, Tongji Hospital, Tongji Medical College, Huazhong University of Science and Technology, Wuhan, 430030, Hubei, China

**Keywords:** SLE, COVID-19, SARS-CoV-2, hydroxychloroquine, multiple infections

## Abstract

The coronavirus disease 2019 (COVID-19) has become a global pandemic, which is induced by infection of severe acute respiratory syndrome coronavirus 2 (SARS-CoV-2). Patients with systemic lupus erythematosus (SLE) are susceptible to infections due to the chronic use of immunosuppressive drugs and the autoimmune disorders. Now we report a case of SLE infected with SARS-CoV-2, influenza A virus and *Mycoplasma pneumoniae* concurrently. The patient used hydroxychloroquine and prednisone chronically to control the SLE. After infection of SARS-CoV-2, she was given higher dose of prednisone than before and the same dosage of hydroxychloroquine. Besides, some empirical treatments such as antiviral, antibiotic and immunity regulating therapies were also given. The patient finally recovered from COVID-19. This case indicated that hydroxychloroquine may not be able to fully protect SLE patient form SARS-CoV-2. Intravenous immunoglobulin therapies and increased dose of corticosteroids might be adoptable for patient with both COVID-19 and SLE. Physicians should consider SARS-CoV-2 virus test when SLE patient presented with suspected infection or SLE flare under the epidemic of COVID-19.

## 1 Introduction

The coronavirus disease 2019 (COVID-19) is induced by infection of severe acute respiratory syndrome coronavirus 2 (SARS-CoV-2) and has become a global pandemic [1]. The most common symptoms were fever and dry cough. Some patients presented with gastrointestinal symptoms such as diarrhea. The most common imaging feature on chest computed tomography (CT) was ground-glass opacity. Lymphopenia with or without leucopenia was the most important laboratory finding [2,3]. Sepsis, acute respiratory distress syndrome, acute cardiac injury, acute kidney injury and secondary infections were common complications in patients with poor outcomes [4].

Systemic lupus erythematosus (SLE) is an autoimmune disease with multiple systematic damages and complex clinical manifestations. SLE patients are more susceptible to infections due to long-term use of immunosuppressive drugs and its autoimmune disorders. Infection may exacerbate the activity of SLE, and it is also a major cause of death among SLE patients accounting for 37.3% [5]. In circumstance of epidemic of COVID-19, SLE patients are at risk of infection with SARS-CoV-2 [6]. The clinical characteristics of lupus flare are similar to COVID-19, and coexist of these two situations may be easily neglected.

Cases infected with SARS-CoV-2 and influenza viruses concurrently had been reported, and the clinical courses of these cases were similar to single infection with SARS-CoV-2 [7]. Here, we report a female SLE patient with chronic use of hydroxychloroquine infected with SARS-CoV-2, influenza A virus and *Mycoplasma pneumoniae* concurrently.

## 2 Case presentation

The patient is a 65-year-old woman with well-controlled SLE. Her long-term treatment for SLE is prednisone (10 mg p.o., once daily) and hydroxychloroquine (0.2 g p.o., twice daily). She lived 6 miles away from Huanan Seafood Wholesale Market where was the earliest epidemic focus of COVID-19 in Wuhan. She had neither been to the market nor closely contacted with confirmed or suspected COVID-19 patients.

On January 22, 2020, the patient presented fever (peak value: 39°C) with chills, rigor, muscular soreness and fatigue. She coughed with small amount of white sputum occasionally. Sometimes, she felt suffocated after movements. The next day, she developed nausea, anorexia, heartburn and diarrhea (liquid stool, three times per day). On January 25, she visited the clinic for the first time and was given oral medicines (oseltamivir 75 mg twice daily, arbidol 0.3 g twice daily, moxifloxacin 0.4 g once daily and Lianhuaqingwen capsules 1.4 g three times daily). The dosage of prednisone and hydroxychloroquine remained the same as her usually used.

After taking the aforementioned drugs for 7 days, the patients’ symptoms did not improve and returned to the clinic. The outpatient doctor took nasopharyngeal swabs to test SARS-CoV-2 and influenza A virus by real-time reverse transcription polymerase chain reaction (rRT-PCR), both of which were positive. At the same time, her thoracic CT showed bilateral patchy ground-glass opacities (according to the medical records, images not available). Hence, human immunoglobulin was given intravenously (IVIg, 5 g once daily) with the aforementioned oral medicines and increased dose of prednisone (25 mg once daily) for 5 days since February 4. Intravenous immunoglobulin is immunoglobulin G made from healthy human plasma, and low dose of IVIg was used for nonspecific and wide-spectrum antiviral and antibacterial therapies.

On February 8, the patient’ symptoms were relieved partially, but the second thoracic CT showed pulmonary lesions extension (according to the medical records, images not available). Laboratory tests found leucocytosis (11.09 × 10^9^/L), neutrophilic leucocytosis (9.46 × 10^9^/L) and normal lymphocyte count (1.33 × 10^9^/L) (Table 1). Then, the patient was admitted to fever ward of Sino-French New City Branch of Tongji Hospital, a designated hospital for diagnosis and treatment of COVID-19 in Wuhan.

**Table 1:** Laboratory findings. The laboratory findings of the patient during hospitalization

Variables	Normal range	D0	D1	D8	D15	D18	D23
		2/8	2/9	2/16	2/23	2/26	3/2
WBC count (×10^9^/L)	3.50–9.50	11.09	8.03	12.17	11.1	11.77	10.7
LYM count (×10^9^/L)	1.10–3.20	1.33	1.44	2.11	2.26	2.6	2.31
NGC count (×10^9^/L)	1.80–6.30	9.46	5.83	9.12	7.68	7.96	7.15
RBC count (×10^9^/L)	3.80–5.10		3.53	3.53	3.46	3.52	3.73
Hemoglobin (g/L)	115.0–150.0		99.0	103.0	100.0	101.0	106.0
Platelet count (×10^9^/L)	125.0–350.0	289.0	389.0	447.0	261.0	244.0	246.0
IgA (g/L)	0.82–4.53		6.75		6.01		4.94
IgG (g/L)	7.51–15.60		20.40		14.70		12.90
C3 (g/L)	0.64–1.39		0.92		0.87		0.82
C4 (g/L)	0.16–0.38		0.22		0.18		0.23
Interleukin-6 (pg/mL)	<7.00		24.95		7.12	7.25	10.67
TNF-α (pg/mL)	<8.1		8.1		6.5	12.5	8.8
CD4+ T cell count (/μL)	550–1440				1,517		1,603
CD8+ T cell count (/μL)	320–1250				252		361
Th/Ts	0.71–2.78				6.01		4.45
Serum ferritin (×10^2^ μg/L)	15–150		605.3	603.9	434.1	396.6	387
hsCRP (mg/L)	<1		79.6		5.7	24.2	39.6
ESR (mm/H)	0.00–20.00		55	72	41	47	47
NT-proBNP (×10^2^ pg/mL)	<285		368		60		
hsTnI (pg/mL)	≤15.6		7.2		9.4		
D-dimer (μg/mL FEU)	<0.5		0.65	0.48	0.39	0.38	0.38
Fibrinogen (g/L)	2.00–4.00		6.37	4.28	3.36	4.42	5.02
ALT (U/L)	≤33		60	43	34	30	31
Creatinine (μmol/L)	45–84		66	76	77	70	82
eGFR (mL/min/1.73 m^2^)	>90		79.6	71.1	70	78.6	64.9
Glycosylated hemoglobin (%)	4.0–6.0		7.1				

Abbreviations: WBC, white blood cells; LYM, lymphocyte; NGC, neutrophilic granulocyte; RBC, red blood cells; IgA, immunoglobin A; IgG, immunoglobin G; TNF-α, tumor necrosis factor α; Th/Ts, helper T cell vs suppressor T cell; hsCRP, high-sensitivity C-reactive protein; ESR, erythrocyte sedimentation rate; NT-proBNP, amino-terminal pro-brain natriuretic peptide; hs-TnI, high-sensitivity cardiac troponin I; ALT, alanine aminotransferase; eGFR, estimated glomerular filtration rate.

**Figure 1:**
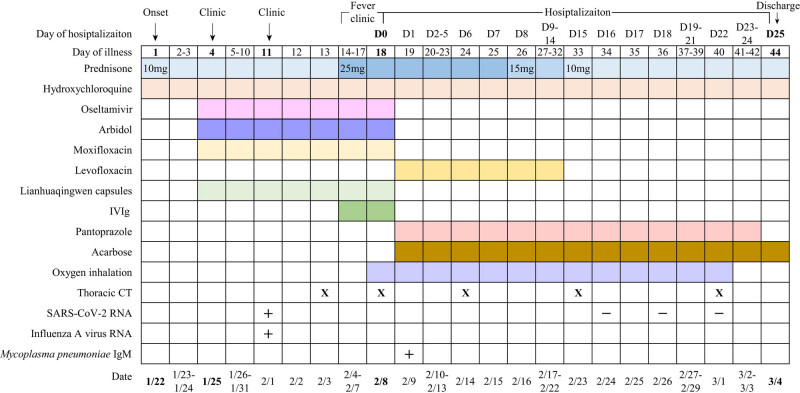
Treatments, radiology and pathogen findings according to day of illness and day of hospitalization. Symbols: X, CT conducted; +, positive result; −, negative result. Abbreviations: IVIg, intravenous immunoglobulin; CT, computed tomography; SARS-CoV-2, severe acute respiratory syndrome coronavirus 2; IgM, immunoglobulin M.

On admission, the patient presented with mild coughing. Physical examination revealed that the conscious of the patient was clear, temperature was 37.1°C, blood pressure was 136/74 mmHg, pulse rate was 77 beats per minute, respiratory rate was 18 breaths per minute and peripheral oxygen saturation under oxygen inhalation (3 L/min) through nasal catheter was 98%. The results of laboratory examinations were presented in Table 1. White blood cell count, neutrophilic granulocyte count and lymphocyte count were normal. Several inflammatory indicators were high. Mild injuries of kidney, myocardium, liver and coagulation function were observed. *M. pneumoniae* infection was found by serology (Figure 1). Glycosylated hemoglobin was 7.1%, and postprandial blood sugar was higher than 11.1 mmol/L. Routine tests of stool and urine samples were normal.

The patient was diagnosed of SLE combined with COVID-19, influenza A, *M. pneumoniae* infection and diabetes mellitus. Prednisone (25 mg p.o., once daily) and hydroxychloroquine (0.2 g p.o., twice daily) were given to cover the concurrence of SLE and COVID-19. Pantoprazole (40 mg p.o., once daily) was used to prevent gastric mucosal injury caused by prednisone. Levofloxacin (0.5 g p.o., once daily) was given to deal with bacterial and *M. pneumoniae* infection. Then, acarbose (50 mg before lunch and dinner daily) was applied to control the blood glucose.

**Figure 2:**
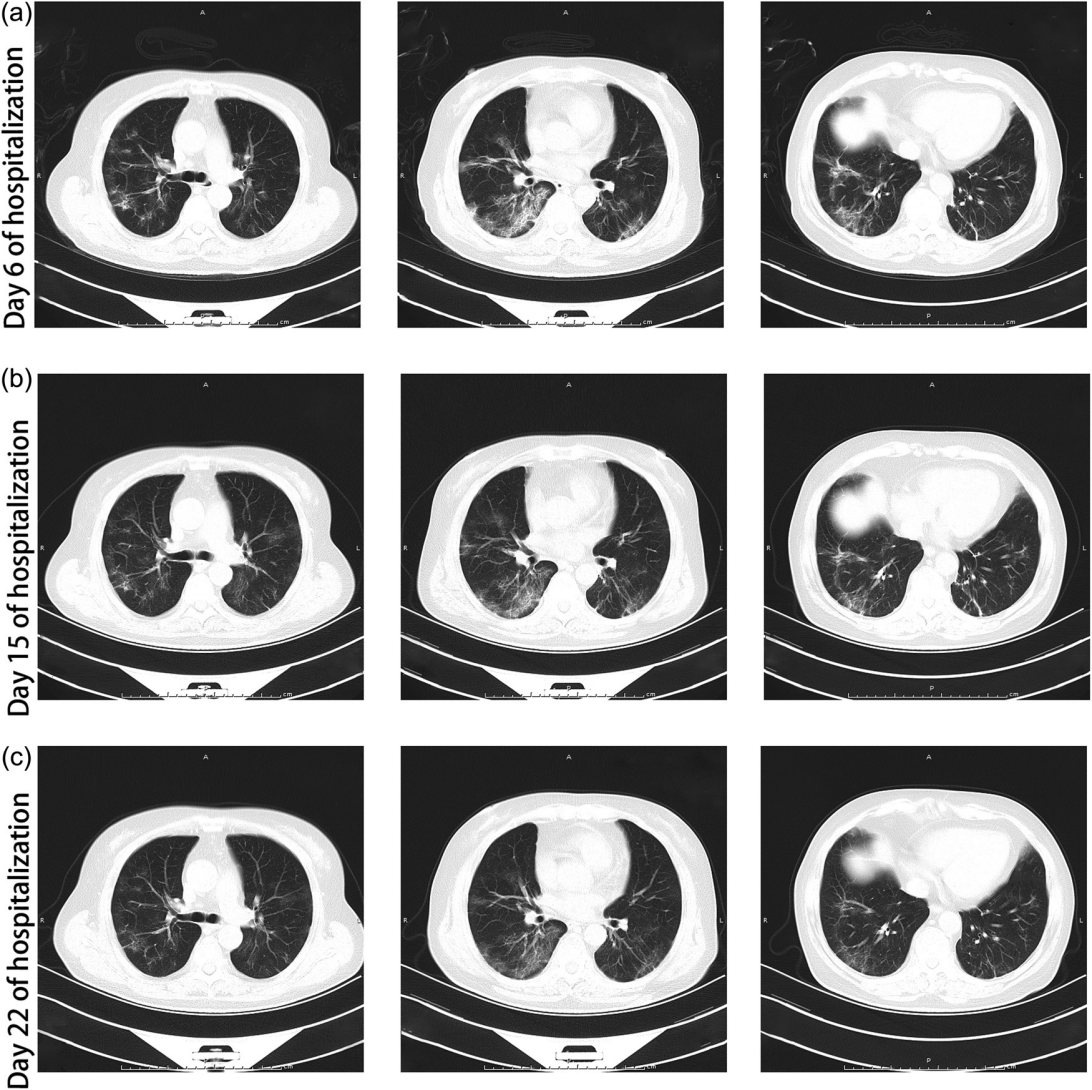
Thoracic CT images. (a) Thoracic CT of day 6 of hospitalization showed bilateral ground-glass, patchy and cord-like shadows. (b) Thoracic CT of day 15 of hospitalization showed that pulmonary infiltration was partly absorbed. (c) Thoracic CT of day 22 of hospitalization showed that pulmonary infiltration was absorbed obviously.

On the 6th day of hospitalization, the symptoms of the patient eased. Thoracic CT (February 14, 2020) showed bilateral ground-glass, patchy and cord-like shadows (Figure 2a), which was better than the second CT (February 8, 2020) before admission (according to the medical records). On the 8th day of hospitalization, some laboratory examinations were retested, and most of which were improved (Table 1). Dosage of prednisone was reduced to 15 mg once daily as pneumonia improved.

On day 15 of hospitalization, laboratory tests revealed that most of the values were better than previous tests (Table 1). Inflammatory indicators such as serum ferritin, high-sensitivity C-reactive protein (hsCRP), erythrocyte sedimentation rate (ESR) and interleukin-6 were lower. Subgroup typing of lymphocytes represented elevation of CD4+ T cells (1517/μL) and reduction of CD8+ T cell (252/μL). The ratio of helper T cell versus suppressor T cell was elevated (6.01). The thoracic CT (February 23, 2020) showed a modest upturn in the lesions (Figure 2b). The dosage of prednisone was then reduced to 10 mg once daily as pneumonia improved. Nasopharyngeal swabs were collected for rRT-PCR of SARS-CoV-2 on day 16 and 18, both of which were negative. Two days later (day 20), the patient developed wrist pain, and loxoprofen (60 mg p.o., twice daily) was used to alleviate it.

On day 22 of hospitalization, no symptoms of the patient were observed. Laboratory findings (Table 1) and thoracic CT (Figure 2c) indicated improvement relative to last time. The rRT-PCR of SARS-CoV-2 of the third nasopharyngeal swab was still negative. Serum immunoglobulin M (IgM) and immunoglobulin G (IgG) of SARS-CoV-2 were 33.73 AU/mL (normal range: ≤10 AU/mL) and 198.77 AU/mL (normal range: ≤10 AU/mL), respectively. Since the patient met the criteria of discharge (temperature came back to normal for over 3 days, respiratory symptoms significantly improved, thoracic radiology showed obvious improvement in acute exudative lesions and negative results of rRT-PCR of SARS-CoV-2 for two nasal pharyngeal swabs over 24 h) [8], she was discharged on March 4, 2020. She has been in good health without any symptoms after discharge.

**Ethical statements:** This study was approved by the Ethics Committee of Tongji hospital, Huazhong University of Science and Technology. All the materials of the patient were anonymous and written informed consent was waived by the ethics committee.

## 3 Discussion

SLE patients are predisposed to infections for long-term immunosuppression, which is a major risk factor of death [5]. Pathogens causing respiratory infection may worsen the activity of SLE, while infection and lupus flare were always easily confused. Since the SLE patient infected with SARS-CoV-2, influenza A virus and *M. pneumoniae*, her symptoms were mixed with all of them. But the symptoms of those diseases were similar to each other, and the mixed symptoms of the patient here represented no big difference from each single disease. This phenomenon was also reported in a case of concurrence of SLE and SARS, which was hard to differentiate between single lupus pneumonitis and mixed diseases when sensitivity of virus nucleic acid detection was not high [9]. Both virus infection and lupus flare may represent fever, fatigue, diarrhea, elevation of ESR and leucopenia. Lupus pneumonitis, virus pneumonia and mycoplasma pneumonia could all showed pulmonary interstitial lesions in radiology. Some biomarkers can help to differentiate lupus activity from infection in SLE patients, such as CRP >6 mg/dL or procalcitonin >0.38 ng/mL [10]. In our case, elevation of white blood cell count and hsCRP may help to identify infections. rRT-PCR of SARS-CoV-2 and influenza viruses was the most important means to confirm virus infections although the sensitivity may not be satisfied. Serology may be false positive, which was due to the cross-reaction of virus antigens and autoantibodies [11]. Most of the laboratory findings of this patient revealed no severe systematic damages except the respiratory system. Her wrist pain developed in the last few days of hospitalization may indicate lupus flare, which was controllable and may be induced by infection. Mathian et al. reported 17 patients with both COVID-19 and SLE (under long-term treatment with hydroxychloroquine). The severe forms of these patients did not seem much different from other COVID-19 cases [6].

So far there are no confirmed effective and widely accepted prophylactic or therapeutic medicines for COVID-19, and most of the treatments were symptomatic and supportive therapies. Chloroquine and hydroxychloroquine are antimalarial agents and are recommended to long-term controlling of SLE activity and had been widely used in clinic for many years. Hydroxychloroquine is a less toxic derivative of chloroquine and had been the first choice of SLE therapy. It was reported that both chloroquine and hydroxychloroquine could inhibit SARS-CoV-2 infection in vitro by blocking the entry stage and post-entry stages of virus invasion [12]. Some study revealed that hydroxychloroquine was more potent than chloroquine to inhibit SARS-CoV-2 *in vitro* [13]. A study based on bioinformatics suggested that chloroquine and hydroxychloroquine may antagonize SARS-CoV-2 by blocking the binding of viral S protein to gangliosides on the host cell surface [14]. Considering the antiviral activity and anti-inflammatory effects of chloroquine and hydroxychloroquine, they were given high expectations [15]. However, a recent cytological study proposed that chloroquine cannot inhibit SARS-CoV-2 entry into lung cells [16]. Maisonnasse et al. established a non-human primate model to evaluate the clinical effects of hydroxychloroquine. They found that hydroxychloroquine had neither antiviral activity nor clinical efficacy, regardless of the timing of treatment initiation [17]. The RECOVERY Collaborative Group reported the preliminary results from a multi-center, randomized, controlled trial of using hydroxychloroquine in hospitalized patients with COVID-19. They found that COVID-19 patients cannot benefit from hydroxychloroquine treatment [18]. The patient in our report received hydroxychloroquine chronically but still infected with SARS-CoV-2, which may not support the prophylactic ability of hydroxychloroquine. The study of 17 patients with both COVID-19 and SLE (under long-term treatment with hydroxychloroquine) by Mathian et al. did not support the prevention or severity alleviation effect of hydroxychloroquine [6].

Corticosteroids are routine medicine for SLE long-term controlling, which could suppress the inflammatory and autoimmune activities. Corticosteroids could also increase the risk of infection, and some researchers proposed gradually tapering doses to 5–7.5 mg per day during this pandemic for patients on long-term corticosteroid therapy [19]. Our case received short-time elevated dose of prednisone (peak dosage 25 mg) after infection of SARS-CoV-2 and influenza A virus. Her pneumonia improved gradually under the elevated dosage of corticosteroid therapy. It may indicate that carefully increasing the dosage of corticosteroid for patient with both SLE and COVID-19 could be an option when treating severe pneumonia. More evidences need to be accumulated. Some immune therapies might have protected effects against SARS-CoV-2, including some other pathogen vaccines (potential cross-resistance to SARS-CoV-2), IVIg and convalescent serum [20]. High dose of IVIg could be shortly used to control severe autoimmune inflammatory rheumatic diseases by blocking Fc-gamma receptors and neutralizing inflammatory cytokines [20]. In our case, IVIg was used for its wide spectrum antiviral and antibacterial functions. It was reported that patients with severe SARS-CoV-2 infection had high risks of thrombosis, and higher anticoagulation targets should be considered [21]. The margin of benefit (preventing thrombotic events) and risk (bleeding) for anticoagulant and antiplatelet may be narrow, and the utilization of these therapies should be careful and based on clinical scenarios [22]. The blood d-dimer and fibrinogen of our reported patient were once slightly elevated and gradually came back to normal. Since no evidence of high risk of coagulation was observed, she was not given anticoagulant and antiplatelet therapies.

SARS-CoV-2 causes an inflammatory cytokine storm in patients, which may lead to acute respiratory distress syndrome or extrapulmonary multiple-organ failure [23]. Both corticosteroids and hydroxychloroquine could suppress inflammatory conditions. Hydroxychloroquine could inhibit production of various proinflammatory cytokines [24]. Interleukin-6 of the patient here was high on admission and reduced gradually, while most of other tested cytokines were normal. The attack on lung was massive, while the damages to other organs were mild. It is reported that CD4+ T cell count was lower in severe COVID-19 patients [25]. Higher CD4+ T lymphocyte count was correlated with shorter time of detoxification for feces of COVID-19 patients [26]. CD4+ T cell count of the patient here was high, which may help to explain the rapid clearance of SARS-CoV-2. Lymphopenia is a risk factor of death in COVID-19 patients [4], and the lymphocyte count of this case was normal during the whole course. These may explain the survival of the patient.

In summary, we report an older patient with concurrent COVID-19, SLE, diabetes mellitus and multiple infections, who finally recovered from the COVID-19.This case indicated that hydroxychloroquine may not be able to fully protect SLE patient form SARS-CoV-2. IVIg therapies and increased dose of corticosteroids might be adoptable for patient with both COVID-19 and SLE. Physicians should consider SARS-CoV-2 virus test when SLE patient presented with suspected infection or SLE flare under the epidemic of COVID-19. The experience of only one case cannot be extrapolated, and more observations of such cases and further investigations are needed in the future.

## Abbreviations

COVID-19: coronavirus disease 2019
SARS-CoV-2: severe acute respiratory syndrome coronavirus 2
SLE: systemic lupus erythematosus
CT: computed tomography
rRT-PCR: real-time reverse transcription polymerase chain reaction
IVIg: intravenous immunoglobulin
hsCRP: high-sensitivity C-reactive protein
ESR: erythrocyte sedimentation rate
IgM: immunoglobulin M
IgG: immunoglobulin G
